# LAMP1 targeting of the large T antigen of Merkel cell polyomavirus results in potent CD4 T cell responses and tumor inhibition

**DOI:** 10.3389/fimmu.2023.1253568

**Published:** 2023-08-30

**Authors:** Claire Buchta Rosean, Erica C. Leyder, Jeneice Hamilton, Joseph J. Carter, Denise A. Galloway, David M. Koelle, Paul Nghiem, Teri Heiland

**Affiliations:** ^1^ Immunomic Therapeutics Inc., Rockville, MD, United States; ^2^ Human Biology Division, Fred Hutchinson Cancer Research Center, Seattle, WA, United States; ^3^ Department of Medicine, University of Washington, Seattle, WA, United States; ^4^ Vaccine and Infectious Diseases Division, Fred Hutchinson Cancer Center, Seattle, WA, United States; ^5^ Department of Laboratory Medicine and Pathology, University of Washington, Seattle, WA, United States; ^6^ Department of Global Health, University of Washington, Seattle, WA, United States; ^7^ Department of Translational Research, Benaroya Research Institute, Seattle, WA, United States; ^8^ Division of Dermatology, Department of Medicine, University of Washington, Seattle, WA, United States; ^9^ Clinical Research Division, Fred Hutchinson Cancer Center, Seattle, WA, United States

**Keywords:** immunotherapy, cancer vaccine, Merkel cell polyomavirus, Merkel cell carcinoma, tumor microenvironment

## Abstract

**Introduction:**

Most cases of Merkel cell carcinoma (MCC), a rare and highly aggressive type of neuroendocrine skin cancer, are associated with Merkel cell polyomavirus (MCPyV) infection. MCPyV integrates into the host genome, resulting in expression of oncoproteins including a truncated form of the viral large T antigen (LT) in infected cells. These oncoproteins are an attractive target for a therapeutic cancer vaccine.

**Methods:**

We designed a cancer vaccine that promotes potent, antigen-specific CD4 T cell responses to MCPyV-LT. To activate antigen-specific CD4 T cells *in vivo*, we utilized our nucleic acid platform, UNITE™ (UNiversal Intracellular Targeted Expression), which fuses a tumor-associated antigen with lysosomal-associated membrane protein 1 (LAMP1). This lysosomal targeting technology results in enhanced antigen presentation and potent antigen-specific T cell responses. LT^S220A^, encoding a mutated form of MCPyV-LT that diminishes its pro-oncogenic properties, was introduced into the UNITE™ platform.

**Results:**

Vaccination with LT^S220A^-UNITE™ DNA vaccine (ITI-3000) induced antigen-specific CD4 T cell responses and a strong humoral response that were sufficient to delay tumor growth of a B16F10 melanoma line expressing LT^S220A^. This effect was dependent on the CD4 T cells’ ability to produce IFNγ. Moreover, ITI-3000 induced a favorable tumor microenvironment (TME), including Th1-type cytokines and significantly enhanced numbers of CD4 and CD8 T cells as well as NK and NKT cells. Additionally, ITI-3000 synergized with an α-PD-1 immune checkpoint inhibitor to further slow tumor growth and enhance survival.

**Conclusions:**

These findings strongly suggest that in pre-clinical studies, DNA vaccination with ITI-3000, using the UNITE™ platform, enhances CD4 T cell responses to MCPyV-LT that result in significant anti-tumor immune responses. These data support the initiation of a first-in-human (FIH) Phase 1 open-label study to evaluate the safety, tolerability, and immunogenicity of ITI-3000 in patients with polyomavirus-positive MCC (NCT05422781).

## Introduction

Merkel cell carcinoma (MCC) is rare type of neuroendocrine skin cancer, affecting ~3000 people annually in the United States (1). MCC presents as a fast-growing nodule, typically on the face, head, or neck. Incidence of MCC increases with age and correlates with long-term sun exposure and immunosuppression (2–5). MCC is aggressive and highly metastatic, with limited treatment options, demonstrating a need for new, targeted therapies for this disease (6). Current therapies for early-stage disease include surgical resection with or without subsequent radiation treatment (7). However, the overall 5-year recurrence rate of MCC is high at ~40% (6, 8–10).

Approximately 80% of MCC cases are associated with Merkel cell polyomavirus (MCPyV) infection, a circular, double-stranded DNA virus that clonally integrates into cells within the skin (2, 4, 11). Infection with MCPyV is common, with the virus ubiquitously detected in the human skin microbiome (11–13). As MCC is a rare cancer type, disease development is clearly multifactorial. MCC cells express viral proteins, including a truncated form of the large T (LT) antigen of MCPyV (14). CD4 T cells from both healthy persons and MCC patients recognize LT, making it an attractive tumor-associated antigen for vaccine development (15). LT functions as an oncoprotein, primarily through N-terminus-mediated sequestration of retinoblastoma protein (pRB). Mutating serine to alanine at position 220 decreases LT binding to pRB, diminishing its pro-oncogenic properties. Human and mouse CD4 T cells recognize both LT^S220A^ and wild-type (WT) LT, suggesting that vaccinating with LT^S220A^ could induce immune responses that target endogenous LT in MCC tumors (16).

A portion of the N-terminal region of LT (the common T region, 78 amino acids) is also used in an alternatively spliced protein (the small T antigen (ST)). Therefore, targeting LT^S220A^ also targets a portion of the other critical MCPyV oncoprotein, ST. Importantly, multiple studies have shown that these viral oncoproteins are obligately expressed in MCC tumors (1). Thus, the fact that viral antigenic loss is not a viable immune escape mechanism in MCC further increases the rationale for the development of a therapeutic vaccine targeting the viral oncoproteins of MCPyV.

We designed a cancer vaccine utilizing our nucleic acid platform, UNITE™ (UNiversal Intracellular Targeted Expression), which fuses a tumor-associated antigen with lysosomal-associated membrane protein 1 (LAMP1). This lysosomal targeting technology results in enhanced antigen presentation and broad adaptive and humoral immune responses (17, 18). LT^S220A^ was introduced into the UNITE™ platform, with the DNA vaccine referred to here as ITI-3000. In addition to driving expression of the LT^S220A^-LAMP1 fusion protein, the plasmid backbone of ITI-3000 contains CpG islands that further activate anti-tumor immune responses through ligation of foreign DNA sensing pathways.

Following injection of UNITE™ vaccines, the glycosylated antigen-LAMP1 fusion protein is expressed in antigen-presenting cells and trafficked to the lysosome. This drives antigen presentation by MHC class II molecules to CD4 T cells, resulting in an antigen-specific Th1 response. The UNITE™ platform also promotes antigen presentation by MHC class I molecules to CD8 T cells (17, 18).

Previous studies using the UNITE™ platform have shown robust antigen-specific humoral and cellular responses triggering significant anti-tumor activity. Therapeutic vaccination with the UNITE™ platform conferred ~50% survival in an orthotopic, syngeneic glioblastoma mouse model (17) and ~30% tumor regression in an endogenous, metastatic HER2^+^ breast cancer model (18). Tumor microenvironment (TME) analyses in these studies demonstrated enhanced activation of CD8 cytotoxic killer T cells and significant CD4 T cell infiltration.

In the present study, we demonstrate that vaccination with ITI-3000 induces potent, antigen-specific CD4 T cell responses and a strong humoral response that were sufficient to delay tumor growth of an orthotopic, syngeneic B16F10 melanoma line expressing LT^S220A^. This effect was dependent on the CD4 T cells’ ability to produce IFNγ. Moreover, ITI-3000 induced a favorable TME, including a Th1-type cytokines and significantly enhanced numbers of CD4 and CD8 T cells as well as NK and NKT cells. Additionally, ITI-3000 synergized with an α-PD-1 immune checkpoint inhibitor (ICI) to further slow tumor growth and enhance survival. These findings strongly suggest that in preclinical studies, DNA vaccination with ITI-3000, using the UNITE™ platform, enhances CD4 T cell responses to MCPyV-LT that result in significant anti-tumor immune responses.

## Materials and methods

### Mice

6-8 week old female C57BL/6J mice and C57BL/6J IFNγ^-/-^ mice were purchased from Jackson Laboratories (Bar Harbor, MA) and maintained at the animal facility at Immunomic Therapeutics (ITI) (Rockville, MD). The animal studies were reviewed and approved by ITI’s Institutional Animal Care and Use Committee (IACUC).

### Vector construction

ITI-3000 is a DNA plasmid where LT^S220A^ is fused with LAMP1 in the NTC8382-VA1 vector. This plasmid was designed as a minimalized antibiotic-free selection vector (Nature Technologies, Lincoln, NE) (19). The vector combines minimal prokaryotic sequences, including a sucrose selectable marker. It also contains a novel chimeric promoter that directs mammalian cell expression. Consistent with the Food and Drug Administration (FDA) regulatory guidance regarding DNA vaccine vector composition, all sequences that were not essential for *Escherichia coli* plasmid replication or mammalian cell expression of the target gene were eliminated. Synthetic eukaryotic mRNA leader and terminators were utilized in the vector to limit DNA sequence homology with the human genome to reduce the possibility of chromosomal integration (20). In addition, the LT^S220A^ sequence was codon optimized to enhance expression. Control vector was generated by ITI (NTC8382-VA1, vector backbone alone without insert). All plasmid lots underwent plasmid form analysis *via* HPLC, endotoxin, osmolality, and gross contamination tests. Expression of the LT^S220A^-LAMP1 fusion protein was confirmed at the correct molecular weight by Western blot (**Supplemental Figure 1**).

### Immunization

Mice were immunized with 40μg of ITI-3000 *via* intradermal (ID) injection in the mouse ear pinna followed by electroporation (EP) using the ICHOR TriGrid Delivery System, model-TDS II (San Diego, CA). For control vector groups, 40μg of control vector plasmid was given ID/EP.

### LT^S220A^-expressing B16F10 cells

B16F10 melanoma cells were purchased from ATCC (Manassas, VA). The B16F10 melanoma cell line expressing LT^S220A^ (B16-LT) was generated by ITI *via* retroviral transduction using a vector containing the sequence for GFP. A clonal population was selected by limited dilution cloning (0.6 cells/well) followed by flow cytometry to confirm clonal GFP expression. Expression of the LT^S220A^ antigen was confirmed by Western blot (**Supplemental Figures 1, 2**).

Tumor cells were injected subcutaneously in the right flank. 2E5 B16-LT cells were injected for prophylactic studies, and 5E4 B16-LT cells were injected for all therapeutic studies. Before injection, cells were maintained for less than four passages, and morphology was monitored. Tumor area was calculated as (width^2^ x length)/2, where width is the smallest measurement. Mice were euthanized when tumors reached a size of 2000mm^3^ or 20mm in any direction, when tumors had ulcerations greater than 2mm, or when mice showed distress or signs of peritoneal tumors, indicating that the tumor had grown through the peritoneal wall.

### Tissue collection and processing

For ELISpot and intracellular cytokine staining (ICS) by flow cytometry, spleens were collected and crushed through a 70μM cell strainer to form a single cell suspension. Red blood cells were lysed (Tonbo Biosciences, San Diego, CA) and splenocytes were counted (Cellometer Auto 2000, Nexcelom Bioscience, Lawrence MA). To analyze the TME, mice were treated with brefeldin A, as previously described (21) (Sigma-Aldrich, St. Louis, MO, 250μg per mouse) for four to six hours before euthanization. Tumors were collected, weighed, processed (Tumor Dissociation Kit and gentleMACS dissociator, Miltenyi Biotec, Germany), and total cells were counted.

### ELISpot

The overlapping peptide library spanning LT-MCPyV (peptide length: 13 amino acids, overlap: 9 amino acids) was synthesized by GenScript (Piscataway, NJ) (22). Peptides were resuspended at 50mg/ml in DMSO, and five peptide pools containing 25 peptides each were created at a final concentration of 2mg/ml. Peptide pool 5 additionally contained a 9-mer peptide previously identified as an optimized H-2K^b^ CD8 T cell epitope (IAPNCYGNI) (23) and two 20-mer peptides previously identified as optimized CD4 T cell epitopes in C57BL/6 mice (PHSQSSSSGYGSFSASQASD and SSSSGYGSFSASQASDQSRG) (24). Working concentration of each peptide in assays was 2μg/ml. Concanavalin A (Sigma-Aldrich), a positive control, was used at 0.125μg/ml for IFNγ ELISpots and at 2.5μg/ml for IL-4 ELISpots. “Media” used was RPMI 1640, 10% FBS, 1% penicillin/streptomycin, 1% L-glutamine, and 0.1% beta-mercaptoethanol. ELISpot plates (Millipore, Burlington, MA) were coated with purified anti-mouse IFNγ (AN-18, Biolegend, San Diego, CA) or purified anti-mouse IL-4 (11B11, Biolegend). After 48 hours of incubation at 37C, plates were developed with biotin-conjugated anti-mouse IFNγ (R4-6A2, Biolegend) or anti-mouse IL-4 (BVD6-24G2, Biolegend), streptavidin-HRP (Becton Dickinson, Franklin Lakes, NJ), and AEC substrate kit (Becton Dickinson). Plates were read on an AID plate reader (Autoimmun Diagnostika, Germany).

### Flow cytometry

For ICS, splenocytes were incubated with brefeldin A (Becton Dickinson) and monensin (Becton Dickinson) for 5 hours at 37C in the presence or absence of peptides overlapping LT-MCPγV. Cells were washed with FACS buffer (PBS, 2% FBS, 2mM EDTA) and stained with Zombie aqua for live/dead discrimination, anti-CD16/32 to block Fc receptors (93, purified), and fluorochrome-conjugated antibodies at the manufacturer’s recommended dilution: CD4 FITC (GK1.5), CD8α PerCP/Cyanine5.5 (53-6.7), CD44 PE/Cy7 (IM7), CD3 Alexa Fluor 700 (17A2), and CD62L BV605 (MEL-14), all from Biolegend. Cells were then fixed (Cytofix/Cytoperm, Becton Dickinson) and stained with fluorochrome-conjugated antibodies against intracellular targets at the manufacturer’s recommended dilution: TNFα APC (MP6-XT22), IFNγ BV421 (XMG1.2), and IL-2 PE (JES6-5H4), all from Biolegend.

For TME analysis, cells were washed with FACS buffer and stained with Zombie aqua for live/dead discrimination, anti-CD16/32 to block Fc receptors (93, purified), and fluorochrome-conjugated antibodies at the manufacturer’s recommended dilution: all antibodies from splenic ICS in addition to: CD45 APC/Fire 750 (30-F11), CD3 FITC (17A2), CD4 APC (GK1.5), CD25 PE/Cy7 (PC61), CD27 PE (LG.3A10), NK1.1 PE/Cy7 (PK136), CD11b APC (M1/70), CD11b FITC (M1/70), and F4/80 BV421 (BM8), all from Biolegend. Cells were then fixed (Cytofix/Cytoperm, Becton Dickinson or eBioscience Foxp3/Transcription Factor Staining Buffer Set, Thermo Fisher Scientific, Waltham, MA; as appropriate) and stained with fluorochrome-conjugated antibodies against intracellular or intranuclear targets, as appropriate, at the manufacturer’s recommended dilution (antibodies from splenic ICS in addition to Foxp3 BV421 (MF-14), Biolegend) (see flow cytometry panels in **Supplemental Table 1**).

Compensation for flow cytometry was done using single-stained UltraComp eBeads (eBioscience). Samples were run on a CytoFLEX flow cytometer (Beckman Coulter, Brea, CA) and analyzed using FlowJo (Becton Dickinson).

### Detection of anti-LT humoral immune responses

Antibody responses to different regions of the LT protein were evaluated by incubating serum with fluorescently-labeled beads linked to GST-fusion proteins (the truncated large T antigen (tLT), composed of the first 258 amino acids of LT, or small T antigen (ST)) as previously described (25). To confirm antibody reactivity to the unique region of LT, serum from ITI-3000-vaccinated mice was pre-treated with soluble GST-common T region (first 78 amino acids of T antigen) before addition of the GST-fusion protein-linked beads. Data was reported as mean fluorescence intensity (MFI).

### Cytokine analysis

Serum was collected at the time of euthanization. Tumors were lysed (MSD Tris lysis buffer with protease inhibitor cocktail, Meso Scale Discovery, Rockville, MD) and homogenized (Minilys homogenizer, CKMix 2ml tubes, Bertin Technologies, France). Protein concentrations were determined by bicinchoninic acid assay (BCA), and 100μg of tumor lysate was used in the V-PLEX Proinflammatory Panel 1 Mouse Kit (Meso Scale Discovery). Plates were read using MESO QuickPlex SQ 120 and analyzed by Discovery Work Bench software (Meso Scale Discovery).

### Multispectral immunohistochemistry

Tumors were excised, fixed in 10% neutral-buffered formalin for 24 hours, and placed in 15% sucrose for 24 hours followed by 30% sucrose with 0.1% sodium azide until processing. Soft-tissue paraffin embedding and slide preparation was done by Histoserv, Inc (Germantown, MD). A murine 5-color panel including CD3, CD4, CD8, FoxP3, and DAPI was done at Ultivue Inc. (Cambridge, MA). Whole-slide images were provided and analyzed with artificial intelligence programming for cell segmentation through Biodock.

### CD4 T cell purification and adoptive transfer

Spleens and cervical lymph nodes (LN) were collected from vaccinated mice. Single-cell suspensions were created using frosted glass slides and pooled within donor groups. Cells were filtered through a 70μM cell strainer, and CD4 T cells were isolated by negative magnetic bead selection (EasySep Mouse CD4^+^ T cell isolation kit, StemCell Technologies, Vancouver, Canada). Purity was verified by flow cytometry (>95%). 10E6 purified CD4 T cells were injected intravenously (IV) into recipient mice.

### α-PD-1 antibody treatment

Anti-mouse PD-1 (CD279) antibody (RMP1-14, rat IgG2a, *InVivo*MAb) and its isotype control (2A3, *InVivo*MAb rat IgG2a anti-trinitrophenol) were purchased from BioXCell (Lebanon, NH). 200μg of the appropriate antibody was injected intraperitoneally (IP), weekly, on the same dates as ITI-3000 vaccination.

### Statistics

P values were generated using Student’s t-tests (unpaired, two-tailed, at 95% confidence interval), one-way ANOVA, two-way ANOVA, or Log-rank (Mantel-Cox) test when appropriate (GraphPad Prism, Boston, MA). Statistical significance is designated when p = <0.05.

## Results

### ITI-3000 slows tumor growth and enhances survival in both prophylactic and therapeutic settings

As previous studies using the UNITE™ platform demonstrated robust anti-tumor immune responses (17, 18), we evaluated the anti-tumor activity of ITI-3000 using both prophylactic and therapeutic vaccination strategies. As the cell of origin of MCC is still under debate (26) and a more physiological model was not available, we utilized B16F10, an aggressive C57BL/6-derived melanoma line, as the parental tumor line. Tumor kinetics and survival were performed using B16F10 cells expressing MCPyV-LT (B16-LT). The B16-LT cell line was generated at ITI *via* retroviral transduction, and expression of the LT^S220A^ antigen was confirmed by Western blot (**Supplemental Figures 1, 2**).

**Figure 1:**
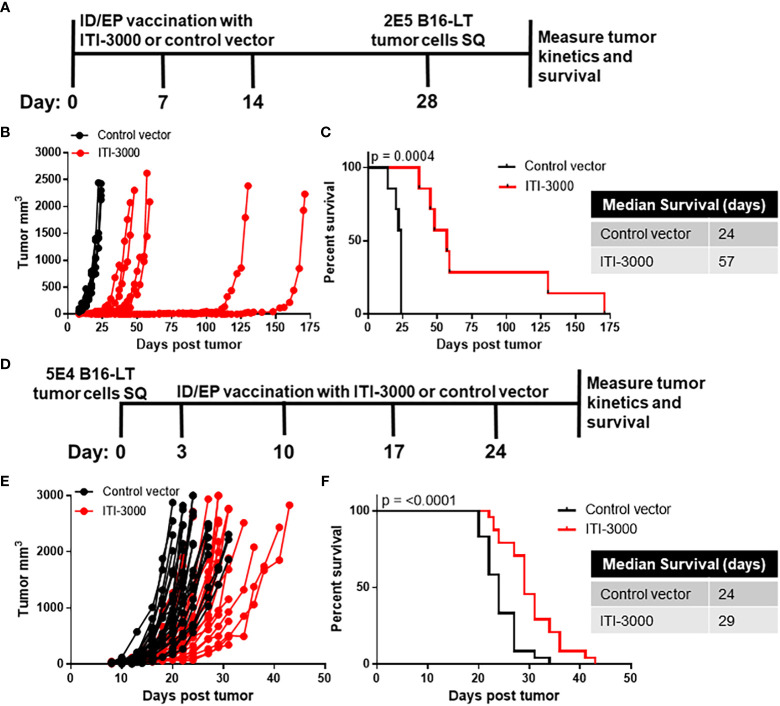
ITI-3000 slows tumor growth and enhances survival in both prophylactic and therapeutic settings. Experimental design for prophylactic vaccination shown in **(A)** C57BL/6 mice were vaccinated three times, weekly, with 40µg of ITI-3000 or control vector *via* intradermal injection followed by electroporation. Fourteen days following the final vaccination, 2E5 B16-LT tumor cells were injected subcutaneously in the right flank. Tumor kinetics **(B)** and survival **(C)** were measured over time. N=7. Representative of three separate experiments. Survival curve significance was calculated using a Log-rank (Mantel-Cox) test. Experimental design for therapeutic vaccination shown in **(D)** C57BL/6 mice were given 5E4 B16-LT tumor cells subcutaneously in the right flank. Starting on day 3 post tumor injection, mice were vaccinated four times, weekly, with 40µg of ITI-3000 or control vector *via* intradermal injection followed by electroporation. Tumor kinetics **(E)** and survival **(F)** were measured over time. N=26 control vector, N=22 ITI-3000, combined data from three experiments. Survival curve significance was calculated using a Log-rank (Mantel-Cox) test.

Experimental designs are shown in **Figure 1A** (prophylactic vaccination) and **Figure 1D** (therapeutic vaccination). The therapeutic model began vaccination on day 3 post tumor implantation, before the tumors were fully established. Although this model is not representative of clinical therapeutic vaccination, it was chosen due to the experimental limitation of a rapidly growing and aggressive mouse tumor such as B16F10. Tumor cells were injected subcutaneously in the right flank. Mice were euthanized when tumors reached a size of 2000mm^3^ or 20mm in any direction, when tumors had ulcerations greater than 2mm, or when mice showed signs of distress. During the in-life portion of the study, no systemic- or location-specific adverse reactions were observed in any treatment group.

Tumor burden was reduced and overall survival was enhanced in ITI-3000-vaccinated mice. Survival curves in the prophylactic setting (**Figure 1C**) demonstrate that the ITI-3000-vaccinated group had a median survival of 57 days, while the control group had a median survival of 24 days. In the therapeutic setting, ITI-3000 vaccination resulted in a significant survival advantage over the control vector with a median survival of 29 days in comparison to the control group at 24 days (**Figure 1F**). Tumor kinetics were also slowed in both the prophylactic and therapeutic settings, as shown in **Figures 1B, E**.

### ITI-3000 induces antigen-specific CD4 T cells and anti-LT antibodies

**Figure 2:**
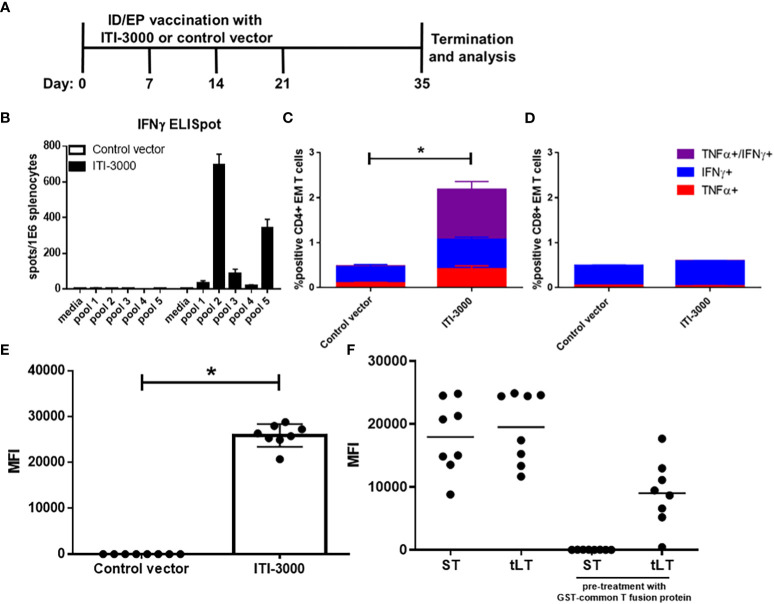
ITI-3000 induces antigen-specific CD4 T cells and anti-LT antibodies. Experimental design shown in **(A)** C57BL/6 mice were vaccinated four times, weekly, with 40µg of ITI-3000 or control vector *via* intradermal injection followed by electroporation. Mice were euthanized fourteen days following the final vaccination, and antigen-specific IFNγ peptide recall responses were evaluated in splenocytes by ELISpot **(B)**. An overlapping peptide library spanning the large T antigen of MCPyV (five separate peptide pools) was used as stimulation at 2µg/ml. Media alone was used as a negative control. Data is represented as spot-forming units (SFU) per 1E6 splenocytes. Representative of three separate experiments. For intracellular cytokine staining (ICS), splenocytes were incubated for five hours in the presence of brefeldin A, monensin, and 2µg/ml LT peptides (peptide pool 2). Cells were then stained with fluorochrome-conjugated antibodies and analyzed using a CytoFLEX flow cytometer. Data is represented as percent cytokine-positive CD4 **(C)** and CD8 **(D)** effector/memory T cells (CD44^+^CD62L^lo^) N=8. Representative of three separate experiments. Statistical significance was calculated by two-way ANOVA. Antibody responses to different regions of the LT protein were evaluated by incubating serum with fluorescently-labeled beads loaded with GST-fusion proteins (the truncated large T antigen (tLT), or small T antigen (ST)). To confirm antibody reactivity to the unique region of LT **(F)**, serum from ITI-3000-vaccinated mice **(E)** was pretreated with soluble GST-common T region before addition of T antigen-loaded beads. N=8. Representative of three separate experiments. Statistical significance was calculated by unpaired Student’s t-test. The "*" symbol represents a p-value of <0.05.

Vaccination with the UNITE™ platform has previously shown robust antigen-specific cellular and humoral responses (17, 18). Using five peptide pools spanning the sequence of MCPyV-LT, we evaluated systemic anti-LT immune responses in naive mice that were vaccinated with ITI-3000 (experimental layout in **Figure 2A**). ELISpot analysis of IFNγ production from total splenocytes demonstrated that mice vaccinated with ITI-3000 showed significant antigen-specific IFNγ responses after stimulation with MCPyV-LT peptides, particularly in response to peptide pool 2 (**Figure 2B**).

To identify the immunodominant region within peptide pool 2, splenic IFNγ responses were tested using each peptide (peptides 1-25) as well as using peptides that were previously-identified as CD4 and CD8 T cell immunodominant epitopes in C57BL/6 mice (23, 24). The majority of the IFNγ spots were produced in response to peptide 13: YGSFSASQASDSQ, corresponding to amino acids 145-157 of the LT protein. This is in agreement with the previously identified CD4 T cell epitopes which spanned amino acids 136-155 and 141-160 of LT (24) (**Supplemental Figure 3**). However, no IFNγ spots were produced in response to the previously identified CD8 T cell epitope.

IL-4 ELISpots showed similar trends, however the number of spots were much lower than those seen in the IFNγ ELISpot (approximately 5x greater IFNγ producing cells) (**Supplemental Figure 4**). This is typical of negative feedback regulation of the Th2 pathway induced in response to strong Th1 activation (27).

To determine the composition of the T cell-mediated immune response after ITI-3000 vaccination, we performed ICS for pro-inflammatory cytokines after stimulation with MCPyV-LT peptides. The flow cytometry gating scheme is shown in **Supplemental Figure 5**. As demonstrated by co-expression of IFNγ and TNFα, antigen-specific effector/memory (EM, CD44^+^CD62L^lo^) CD4 T cells were significantly induced by vaccination with ITI-3000, suggestive of a pro-inflammatory Th1 response (**Figure 2C**). In agreement with ELISpot data in **Supplemental Figure 3**, antigen-specific CD8 EM T cells were not significantly induced by ITI-3000 (**Figure 2D**).

Approximately 50% of MCC patients with clinically detectable disease produce antibodies against the ST antigen of Merkel cell polyomavirus (28). Anti-ST antibody titers correlate with tumor burden and sharply decrease in post-treatment patients with no evidence of active disease (NEAD) (28). Serial testing for increased titers of anti-ST antibodies, known as the Anti Merkel Cell Panel (AMERK), is one standard of care (SOC) method used to detect disease recurrence (25, 28). To confirm an antigen-specific humoral response to the LT antigen, antibody reactivity was evaluated by incubating serum with fluorescently-labeled beads loaded with GST-fusion proteins (the ST antigen, as in the AMERK test, or the truncated LT antigen (tLT)) (25).

As shown as quantification of MFI in **Figure 2E**, serum from ITI-3000-vaccinated mice showed robust antibody reactivity to the tLT antigen in comparison with that observed in control serum. The LT and ST antigens share an N-terminal, 78 amino acid common T region. Thus, we also determined antibody reactivity to unique regions of ST and LT antigens. Serum from ITI-3000-vaccinated mice was pretreated with soluble GST-common T fusion protein to block antibody binding to the common T region, prior to the addition of T antigen protein-loaded beads. As shown in **Figure 2F**, vaccination with ITI-3000 induced antibodies that reacted strongly to both ST and LT, but failed to react to ST when pretreated with GST-common T. This demonstrated that ITI-3000 induced humoral responses that recognize both common T and unique regions of the LT antigen, but not the unique portion of ST.

### ITI-3000 induces immune cell infiltration into tumors

**Figure 3:**
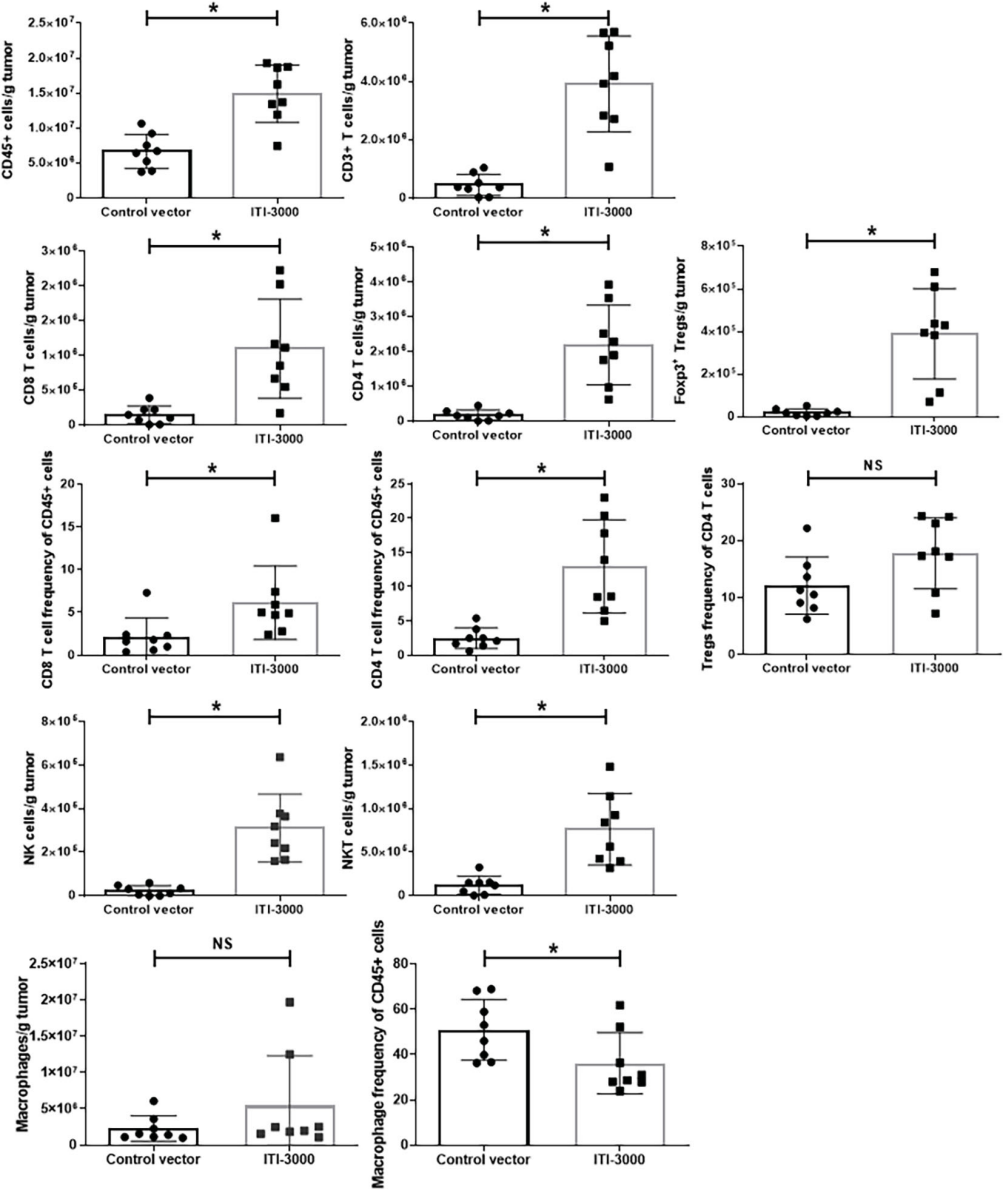
ITI-3000 induces immune cell infiltration into tumors. Mice were given 5E4 B16-LT tumor cells subcutaneously in the right flank. Starting on day 3 post tumor injection, mice were vaccinated twice, weekly, with 40µg of ITI-3000 or control vector *via* intradermal injection followed by electroporation. Three days following the second vaccination, mice were euthanized, and tumors were enzymatically digested and processed into a single cell suspension. The cellular composition of the tumor microenvironment, including numbers of CD45^+^ cells and CD3^+^ cells **(A)**, numbers **(B)** and frequencies **(C)** of CD4^+^, CD8^+^, and CD4^+^Foxp3^+^CD25^+^ T cells, numbers of NK and NKT cells **(D)**, and numbers and frequency of F4/80+ macrophages **(E)**, was analyzed by flow cytometry. Data is represented as total number of cells per gram tumor tissue or as frequency of total CD45^+^ or CD4^+^ cells. N=8. Representative of two separate experiments. Statistical significance was calculated by unpaired Student’s t-test. The "*" symbol represents a p-value of <0.05. "NS" denotes a non-significant difference between groups.

Previous studies found that the therapeutic benefit of the UNITE™ platform is associated with enhanced tumor-infiltrating CD4 and CD8 T cells (17, 18). We investigated the TME after therapeutic vaccination with ITI-3000 to elucidate the mechanism of action (MOA) of ITI-3000. Flow cytometry gating schemes for the T cell, Treg, NK cell, and macrophage panels are shown in **Supplemental Figures 4–8**. **Figure 3** shows the cellular composition of the TME with and without prior ITI-3000 vaccination. Within treated tumors, there was a significant increase in the number of CD45^+^ hematopoietic cells and CD3^+^ T cells in comparison with control tumors (**Figure 3A**). Upon closer inspection of the CD45^+^CD3^+^ population, and in agreement with previous work, there was also an increase in the number of tumor-infiltrating CD4^+^ and CD8^+^ T cells from ITI-3000-treated animals, both in cells per gram tumor (**Figure 3B**) and as frequency of total CD45^+^ cells (**Figure 3C**).

We also saw a significant increase in CD4^+^Foxp3^+^CD25^+^ regulatory T cells (Tregs) in tumors from vaccinated animals (**Figure 3B**). However, the frequency of Tregs of total CD4 T cells was unchanged between groups (**Figure 3C**). Due to this fact, the significant increase in Tregs in tumors from ITI-3000-vaccinated mice likely reflects the increase in total CD4^+^ T cells after vaccination.

We also observed higher numbers of tumor-infiltrating NK and NKT cells in vaccine-treated tumors (**Figure 3D**). Thus, therapeutic vaccination with ITI-3000 induced significantly increased numbers of tumor-infiltrating immune cells that are correlated with anti-tumor function (immunologically “hot” tumors). Corresponding with a “hot” TME, tumors from ITI-3000-vaccinated mice did not show an increase in total numbers of CD11b^+^F4/80^+^ macrophages (immunosuppressive tumor-associated macrophages (TAM)), and the frequency of TAMs was also reduced in the TME after vaccination (**Figure 3E**).

### ITI-3000 induces systemic Th1-type cytokines with enhanced pro-inflammatory cytokines in the TME

**Figure 4:**
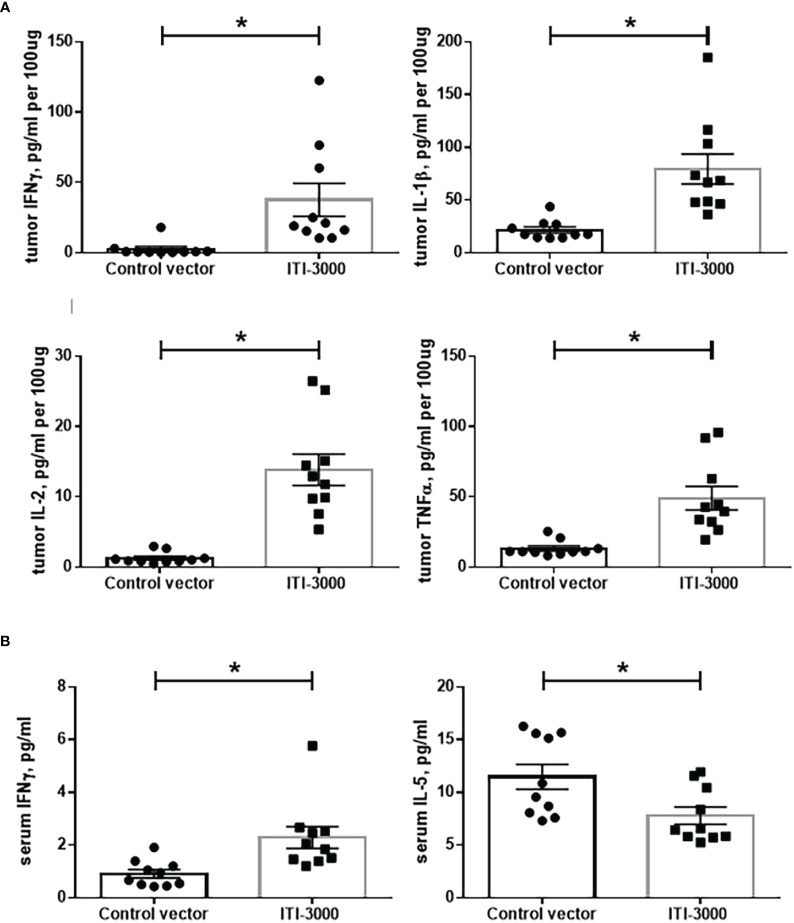
ITI-3000 induces a systemic Th1-type cytokine profile with enhanced pro-inflammatory cytokines in the tumor microenvironment. Mice were given 5E4 B16-LT tumor cells subcutaneously in the right flank. Starting on day 3 post tumor injection, mice were vaccinated twice, weekly, with 40µg of ITI-3000 or control vector *via* intradermal injection followed by electroporation. Three days following the second vaccination, mice were euthanized, and serum and tumors were collected. Tumors were lysed and homogenized, and cytokine content of tumor extracts **(A)** and serum **(B)** was analyzed using the Meso Scale Discovery V-PLEX Proinflammatory Panel 1 Mouse Kit. N=10. Data is combined from two separate experiments. Statistical significance was calculated by unpaired Student’s t-test. The "*" symbol represents a p-value of <0.05.

Preferential activation of Th1 cells expressing pro-inflammatory cytokines after UNITE™ vaccination has been shown previously (17, 18). We evaluated both systemic (serum, **Figure 4B**) and TME-associated (**Figure 4A**) cytokine content after therapeutic vaccination with ITI-3000 using the Meso Scale Discovery V-PLEX Proinflammatory Panel 1 Mouse kit. Pro-inflammatory cytokines such as IFNγ, IL-1β, IL-2, and TNFα were significantly upregulated in ITI-3000-treated tumors (**Figure 4A**). We also found cytokines indicative of a systemic Th1 response in serum from vaccine-treated mice, including significantly upregulated IFNγ and decreased IL-5 (**Figure 4B**). Supporting our observation that ITI-3000 induces antigen-specific CD4 T-cells, ITI-3000 induced Th1-type cytokines both systemically and within the TME.

### ITI-3000 induces spatial T cell infiltration into tumors

**Figure 5:**
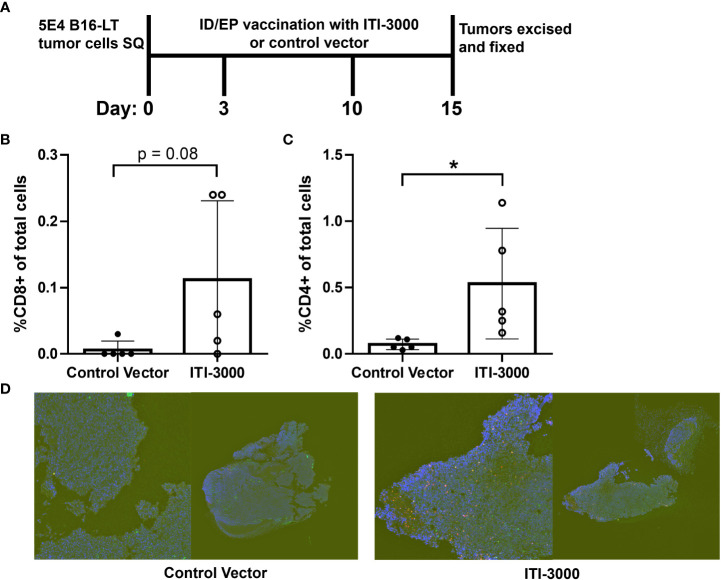
ITI-3000 induces spatial immune T cell infiltration into tumors. Experimental design shown in **(A)**. C57BL/6 mice were given 5E4 B16-LT tumor cells subcutaneously in the right flank. Starting on day 3 post tumor injection, mice were vaccinated twice, weekly, with 40µg of ITI-3000 or control vector *via* intradermal injection followed by electroporation. Tumors were collected and formalin fixed on day 15 post tumor injection, followed by a sucrose gradient, paraffin embedding, and sectioning. The cellular composition of the tumor microenvironment on FFPE slides was determined by multiplex-IHC including percent of CD3^+^ (green), CD8^+^ (magenta) **(B)**, and CD4^+^ (red) **(C)** cells. One representative section (displayed both as whole tumor section (right) and magnified area of interest (left)) of each group is shown in **(D)** The total cell population was determined by nuclear DAPI staining (blue). Sections were analyzed by AI gating in the Biodock platform. N=5. Statistical significance was calculated by unpaired Student’s t-test. The "*" symbol represents a p-value of <0.05.

TME analysis by flow cytometry demonstrated a significant increase in immune cell infiltration after therapeutic vaccination with ITI-3000. To provide greater insight into the spatial organization of these immune cells, we performed multiplex immunohistochemistry (mIHC) in tumors from ITI-3000-vaccinated mice. The experimental design for therapeutic vaccination is shown in **Figure 5A**. The mIHC stain included fluorescent antibodies against CD3, CD4, and CD8. Using whole slide imaging for spatial analysis, we found that the fluorescently-labeled immune cells were located within the confines of soft tumor tissue, indicating T cell infiltration after vaccination with ITI-3000 (**Figure 5D**). Supporting the TME analysis by ICS, the percent of CD3^+^CD4^+^ T cells of the total cell population was increased in animals vaccinated with ITI-3000 as seen in **Figure 5C**, while the slight increase in the percent of CD3^+^CD8^+^ T cells of the total cell population trended toward significance (**Figure 5B**).

### ITI-3000-mediated tumor control is dependent on IFNγ production by antigen-specific CD4 T cells

As shown in previous figures, ITI-3000 induced potent antigen-specific CD4 T cell responses as measured by the production of IFNγ and TNFα. Therefore, we wanted to determine the role of CD4 T cell-mediated cytokine production in the MOA of ITI-3000. As IFNγ is known to be important in CD4 T cell-mediated immune responses (29), we tested the impact of ITI-3000 vaccination in IFNγ^-/-^ mice. We first confirmed that IFNγ^-/-^ mice can mount a sufficient anti-MCPyV-LT immune response in the absence of IFNγ. **Supplemental Figure 9** demonstrates that after vaccination with ITI-3000, CD4 T cells from IFNγ^-/-^ mice produce significantly enhanced TNFα in response to MCPyV-LT peptides, while their production of IFNγ, as expected, was zero.

**Figure 6:**
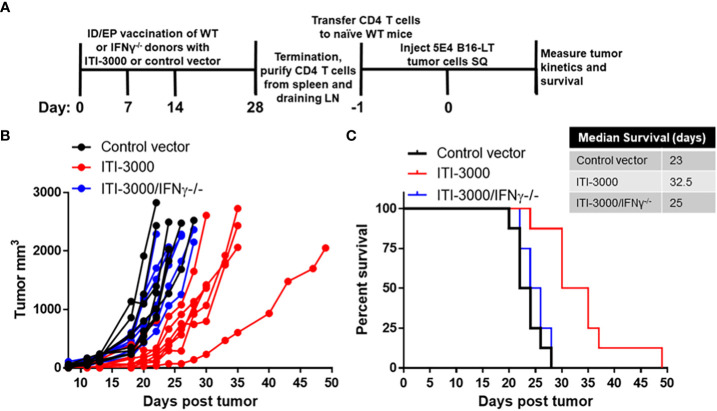
ITI-3000-mediated tumor control is dependent on IFNγ production by antigen-specific CD4 T cells. Experimental design shown in **(A)**. C57BL/6 donor mice (WT or IFNγ^-/-^) were vaccinated three times, weekly, with 40µg of ITI-3000 or control vector *via* intradermal injection followed by electroporation. Mice were euthanized fourteen days following the final vaccination, and total CD4 T cells were purified by magnetic bead negative selection from spleens and draining cervical LN, pooled within groups. 10E6 CD4 T cells, either WT or IFNγ^-/-^, were transferred IV into naïve WT C56BL/6 recipient mice. One day following CD4 T cell transfer, 5E4 B16-LT tumor cells were injected subcutaneously in the right flank. Tumor kinetics **(B)** and survival **(C)** were measured over time. N=8. Representative of two separate experiments. Survival curve significance was calculated using a Log-rank (Mantel-Cox) test corrected for multiple comparisons. Control vector vs ITI-3000 p = 0.0003, ITI-3000 vs ITI-3000/IFNγ^-/-^ p = 0.0007, Control vector vs ITI-3000/IFNγ^-/-^ p = 0.29.

To test the role of IFNγ production by CD4 T cells, naïve WT or IFNγ^-/-^ C57BL/6 mice were vaccinated with ITI-3000 as shown in **Figure 6A**. CD4 T cells were then purified from the spleens and cervical draining LN of ITI-3000- and control-vaccinated mice and IV transferred to naïve WT recipient mice. One day following adoptive cell transfer, B16-LT tumor cells were injected subcutaneously, and tumor kinetics and overall survival was measured over time.

100% of mice that received CD4 T cells from ITI-3000-immunized WT mice showed delayed tumor kinetics and enhanced survival (p = 0.0003). However, mice that received CD4 T cells from ITI-3000-immunized IFNγ^-/-^ mice showed tumor kinetics and overall survival equivalent to that of mice that received CD4 T cells from control-immunized mice (**Figures 6B, C**). These data confirm that antigen-specific CD4 T cells induced by vaccination with ITI-3000 are sufficient to delay tumor progression in the B16-LT model and also identify IFNγ as the key factor involved in this CD4 T cell-mediated mechanism.

### ITI-3000 synergizes with α-PD-1 ICI therapy to further slow tumor growth and enhance survival

**Figure 7:**
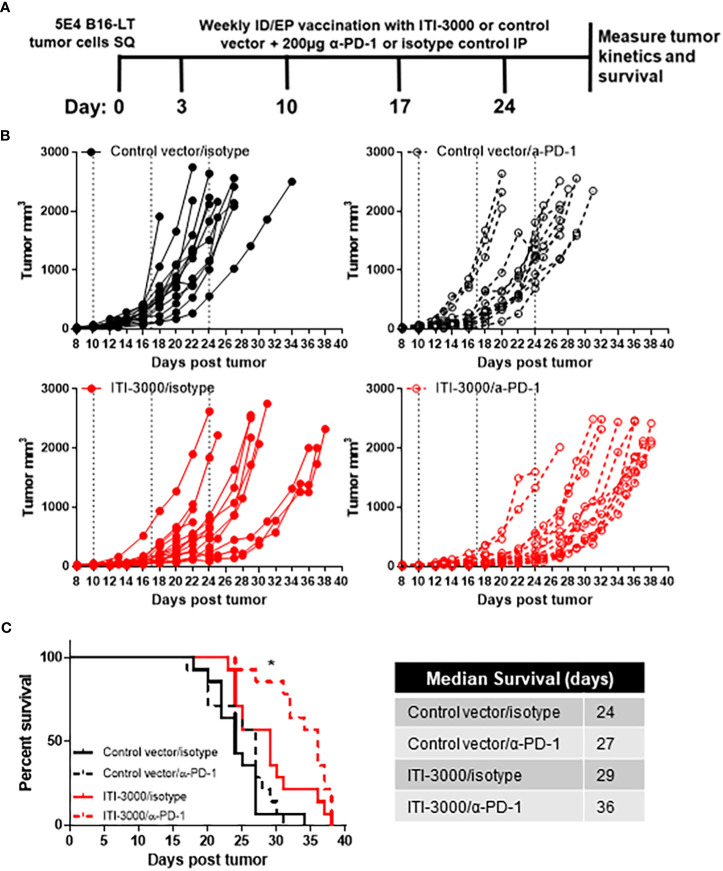
ITI-3000 synergizes with α-PD-1 checkpoint blockade therapy to further slow tumor growth and enhance survival. Experimental design shown in **(A)**. C57BL/6 mice were given 5E4 B16-LT tumor cells subcutaneously in the right flank. Starting on day 3 post tumor injection, mice were vaccinated four times, weekly, with 40µg of ITI-3000 or control vector *via* intradermal injection followed by electroporation. On the same dates, mice were also given 200µg of either α-PD-1 monoclonal antibody or of an isotype control. Tumor kinetics **(B)** and survival **(C)** were measured over time. N=14. Combined data from two experiments. Survival curve significance was calculated using a Log-rank (Mantel-Cox) test corrected for multiple comparisons. Control vector/isotype vs ITI-3000/isotype p = 0.0153, Control vector/isotype vs ITI-3000/α-PD-1 p = <0.0001, ITI-3000/isotype vs ITI-3000/α-PD-1 p = 0.0267. The "*" symbol represents a p-value of <0.05.

Pembrolizumab (30) (α-PD-1) and avelumab (31, 32) (α-PD-L1) are approved for the treatment of advanced MCC. These ICI show an impressive response rate of ~50% in MCC, with relatively durable responses (30–33). One current focus of cancer immunotherapy research is in developing treatments that exhibit synergism with ICI therapies (34). As ITI-3000 modulates the TME toward a “hot” tumor phenotype, we tested combination therapy of ITI-3000 and α-PD-1 ICI in the therapeutic B16-LT tumor model (**Figure 7A**). The α-PD-1 antibody clone chosen for these experiments (RMP1-14) has been shown to block the binding of mouse PD-L1 and PD-L2 to PD-1 and is widely used in mouse models of cancer immunotherapy (35).

Tumor growth was slowed in groups receiving ITI-3000, with animals receiving combination therapy with α-PD-1 antibody showing the greatest reduction in tumor burden (**Figure 7B**). Overall survival was significantly enhanced in ITI-3000-vaccinated mice, with a median survival of 29 days in ITI-3000-vaccinated mice as compared with 24 days in control-vaccinated mice. Combination therapy with α-PD-1 antibody further enhanced survival, with a median survival of 36 days (**Figure 7C**). Survival curve significance was calculated using a Log-rank (Mantel-Cox) test corrected for multiple comparisons. Control vector/isotype vs ITI-3000/isotype p = 0.0153, Control vector/isotype vs ITI-3000/α-PD-1 p = <0.0001, ITI-3000/isotype vs ITI-3000/α-PD-1 p = 0.0267.

In this experimental design, all therapies ceased at day 24 post tumor injection, suggesting that additional treatments may further enhance survival. Thus, these data demonstrate that ITI-3000 synergizes with PD-1 blockade to slow tumor growth and enhance survival.

## Discussion

DNA vaccines incorporating a variety of antigen sequences have been studied in a wide breadth of animal and clinical applications, including infectious disease, cancer, and allergy (17, 18, 36–39). These studies reported induction of low immune responses through major histocompatibility complex (MHC) class 1 molecules without any significant safety concerns. Inclusion of the LAMP1 DNA sequence, linked to the antigen DNA sequence in a bacterial DNA vaccine vector, induces preferential CD4 T cell-mediated immune responses through the MHC class II-lysosome pathway by targeting the antigen to the lysosome (17, 18, 40). Using this UNITE™ vaccine platform, we observe robust adaptive as well as humoral immune responses. The generation of this broad immune response is optimal for the therapeutic treatment of cancers such as Merkel cell carcinoma (MCC).

Currently, there are no treatments for MCC that directly target antigens of MCPyV. As ~80% of MCC cases are driven, in part, by continuing expression of the T oncoprotein (4, 11), this represents an unmet therapeutic need for MCC patients. To address this, we developed ITI-3000, a plasmid DNA vaccine containing the tLT antigen of MCPyV fused with LAMP1. The sequence of the LT antigen contains a single nucleotide substitution of serine to alanine at position 220 which has been previously shown to diminish the oncogenicity of the LT antigen through mutation of the retinoblastoma protein binding site (16). Previous work by another group tested DNA vaccines encoding MCPyV-tLT (24) or ST (41). However, these vaccines did not utilize the UNITE™ platform and were never tested in a clinical trial.

Following administration of ITI-3000, the UNITE™ DNA plasmid is internalized by cells in the target tissue, resulting in expression of glycosylated LT-LAMP1 fusion protein. When this occurs in antigen-presenting cells, the fusion protein is trafficked to the lysosome *via* LAMP1. This drives antigen presentation *via* MHC class II molecules to CD4 T cells, resulting in a robust, antigen-specific Th1 response. The UNITE™ platform also promotes antigen presentation by MHC class I molecules to CD8 T cells (17, 18). However, significant numbers of antigen-specific CD8 T cells were not observed after vaccination with ITI-3000 (**Figure 2D**). This agrees with previous studies using human PBMC and MCC tumor-infiltrating cells that demonstrate strong CD4 T cell-mediated immune responses to the LT antigen (16, 24, 42, 43).

To test anti-tumor efficacy of ITI-3000, we developed an orthotopic, syngeneic MCC model expressing tLT. As the cell of origin of MCC is still under debate (26), we utilized B16F10, an aggressive C57BL/6-derived melanoma line, as the parental tumor line. In both prophylactic (**Figure 1A**) and therapeutic (**Figure 1B**) models of vaccination, ITI-3000 enhanced survival in the B16-LT tumor model. In examining the MOA of ITI-3000, we found that vaccination enhanced systemic antigen-specific immune responses against the LT antigen, including significant CD4 T cell (**Figure 2C**) and anti-LT antibody responses (**Figures 2E, F**).

In evaluation of the TME, we found that ITI-3000 induced tumor infiltration of CD4 and CD8 T cells, measured both by flow cytometry (**Figure 3B**) and by mIHC (**Figures 5B, C**). Cytokine analysis of tumor extracts additionally showed that vaccination with ITI-3000 induced significant expression of IFNγ, IL-1β, IL-2, and TNFα (**Figure 4A**), indicative of a strong anti-tumor immune response. Cytokine expression was critical in the MOA of ITI-3000 as CD4 T cell-mediated IFNγ production was essential for the anti-tumor immune response induced by vaccination (**Figure 6**).

Canonically, CD8 T cells are considered the main player in cancer immunotherapy. However, as demonstrated in this study, CD4 T cells can mount effective anti-tumor immune responses. Potential mechanisms include induction of tumoricidal macrophages, assisting CD8 cytotoxic T cells, and even direct cytolytic killing of tumor cells (44, 45). Indeed, we have previously shown that CD4 T cell activation is involved in the stimulation of cytotoxic CD8 T cells (18). While some cancers do not express MHC class II molecules and thus cannot be directly recognized by CD4 T cells, human melanoma, MCC, and murine B16F10 cells can express MHC class II molecules, with upregulated expression observed in the presence of IFNγ (46, 47). Therefore, it is possible that antigen-specific CD4 T cells induced by ITI-3000 vaccination exhibit direct cytolytic activity in the B16-LT tumor model.

Alternatively, IFNγ, and potentially other soluble factors, produced by vaccine-induced CD4 T cells may impact additional signaling pathways within tumors or other immune cell types to augment tumor killing. One such potential pathway is IFNγ-mediated upregulation of SOCS1 in tumor cells. SOCS1 binds growth factor receptors and inhibits signaling through downstream receptor tyrosine kinases, sensitizing tumor cells to apoptosis and direct killing (48, 49). Future studies will explore these possibilities to further define the MOA of ITI-3000.

ICI, such as monoclonal antibodies blocking the interaction of PD-1 with its ligand PD-L1, have revolutionized cancer treatment by reinvigorating functionally-exhausted T cells within the TME (50, 51). Pembrolizumab (30) (α-PD-1) and avelumab (31, 52) (α-PD-L1) are approved for the treatment of metastatic MCC, with nivolumab (α-PD-1) currently being evaluated in clinical trials (53). These ICI show an impressive response rate of ~50% in MCC, with relatively durable responses (31–33, 54). However, there is still an unmet need for new therapies in the ~50% of patients that do not respond to ICI and in those who experience partial response or disease progression following an initial response. In this study, we show that ITI-3000 synergizes with α-PD-1 ICI (**Figure 7**), potentially through vaccine-mediated influx of antigen-specific T cells into the TME, making ITI-3000 a promising candidate for future combination therapies.

SOC therapy for early-stage and locally-advanced MCC is surgical resection with or without subsequent radiation treatment (7). Despite the high recurrence rate (6, 8, 9), there are currently no approved therapies for MCC patients following surgical resection. Instead, patients are managed by “watchful waiting:” clinical observation which may include regular physical exams, anti-MCPyV antibody screening (known as the AMERK test) (25), and imaging including computed tomography scans to detect potential recurrence. Adjuvant ICI are currently being tested as alternatives to SOC observation: pembrolizumab in patients with completely resected stage I-IIIb MCC in the Surgically Treated Adjuvant Merkel Cell Carcinoma with Pembrolizumab (STAMP) trial (55) (NCT03712605), and avelumab in resected stage III, LN-positive MCC in the Adjuvant Avelumab in Merkel Cell Cancer (ADAM) trial (NCT03271372), among other ongoing trials. ITI-3000 may be an alternative, reduced-toxicity option to adjuvant ICI therapies or could be used in combination with pembrolizumab/avelumab, to enhance recurrence free survival in the adjuvant setting. Of note, the increased humoral response seen after vaccination with ITI-3000 has important clinical implications, as the AMERK antibody test would be unreliable as a marker of potential tumor recurrence in this setting.

In summary, these findings strongly suggest that in pre-clinical studies, DNA vaccination with ITI-3000, using the UNITE™ platform, enhances CD4 T cell responses to MCPyV-LT. Vaccination results in significant anti-tumor immune responses, making ITI-3000 a promising new therapy for treatment of MCPyV-positive Merkel cell carcinoma, either as a monotherapy or in combination with ICI treatment. These data support the initiation of a first-in-human (FIH) Phase 1 open-label study to evaluate the safety, tolerability, and immunogenicity of ITI-3000 in patients with polyomavirus-positive MCC (NCT05422781).

## Data availability statement

The raw data supporting the conclusions of this article will be made available by the authors, without undue reservation.

## Ethics statement

The animal study was approved by ITI’s Institutional Animal Care and Use Committee (IACUC). The study was conducted in accordance with the local legislation and institutional requirements.

## Author contributions

CB: Conceptualization, Formal Analysis, Investigation, Methodology, Writing – original draft, Writing – review & editing. EL: Formal Analysis, Investigation, Writing – original draft, Writing – review & editing. JH: Formal Analysis, Investigation, Writing – review & editing. JC: Formal Analysis, Investigation, Writing – review & editing. DG: Supervision, Writing – review & editing. DK: Conceptualization, Methodology, Writing – review & editing. PN: Conceptualization, Methodology, Writing – review & editing. TH: Conceptualization, Methodology, Supervision, Writing – review & editing.

## Glossary

**Table:**

ADAM	Adjuvant Avelumab in Merkel Cell Cancer
AEC	3-amino-9-ethylcarbazole
AMERK	anti-Merkel cell panel
ANOVA	analysis of variance
B16-LT	B16F10 melanoma cells transduced with MCPyV-LT^S220A^
BCA	bicinchoninic acid assay
CD	cluster of differentiation
CpG	cytosine-phosphorothioate-guanine
DAPI	4′,6-diamidino-2-phenylindole
DMSO	dimethylsulfoxide
DNA	deoxyribonucleic acid
ELISpot	enzyme-linked immunosorbent spot
EM	effector/memory
EP	electroporation
FDA	Food and Drug Administration
FIH	first in human
GFP	green fluorescent protein
GST	glutathione S-transferase
HER2	human epidermal growth factor receptor 2
HPLC	high-performance liquid chromatography
HRP	horseradish peroxidase
IACUC	Institutional Animal Care and Use Committee
ICI	immune checkpoint inhibitor
ICS	intracellular cytokine staining
ID	intradermal
IL	interleukin
IP	intraperitoneal
ITI	Immunomic Therapeutics Inc.
IFNγ	interferon gamma
ITI-3000	DNA vaccine encoding a mutated form of MCPyV-LT and LAMP1
IV	intravenous
LAMP1	lysosomal-associated membrane protein 1
LN	lymph node
LT	large T antigen
mRNA	messenger ribonucleic acid
MCC	Merkel cell carcinoma
MCPyV	Merkel cell polyomavirus
MFI	mean fluorescence intensity
MHC	major histocompatibility complex
mIHC	multispectral immunohistochemistry
mm	millimeter
MOA	mechanism of action
NEAD	no evidence of active disease
NK	natural killer
PBMC	peripheral blood mononuclear cells
PD-1	programmed death ligand 1
pRB	retinoblastoma protein
S220A	mutation of serine to alanine at amino acid position 220
SOC	standard of care
SOCS1	suppressor of cytokine signaling-1
ST	small T antigen
STAMP	Surgically Treated Adjuvant Merkel Cell Carcinoma with Pembrolizumab
TAM	tumor-associated macrophage
Th	T helper
TLR	Toll-like receptor
tLT	truncated large T antigen
TME	tumor microenvironment
TNFα	tumor necrosis factor alpha
Treg	regulatory T cell
μg	microgram
μM	micrometer
UNITE ™	Universal Intracellular Targeted Expression
WT	wild type
