# Supplementary material for: LAMP1 targeting of the large T antigen of Merkel cell polyomavirus results in potent CD4 T cell responses and tumor inhibition
Source: Front Immunol. 2023 Aug 30;14:1253568. doi: 10.3389/fimmu.2023.1253568 (PMC10499392; doi:10.3389/fimmu.2023.1253568)
Supplement: Supplementary file 1 [file DataSheet_1.pdf]

Supplementary Material

1.1 Supplementary Figures

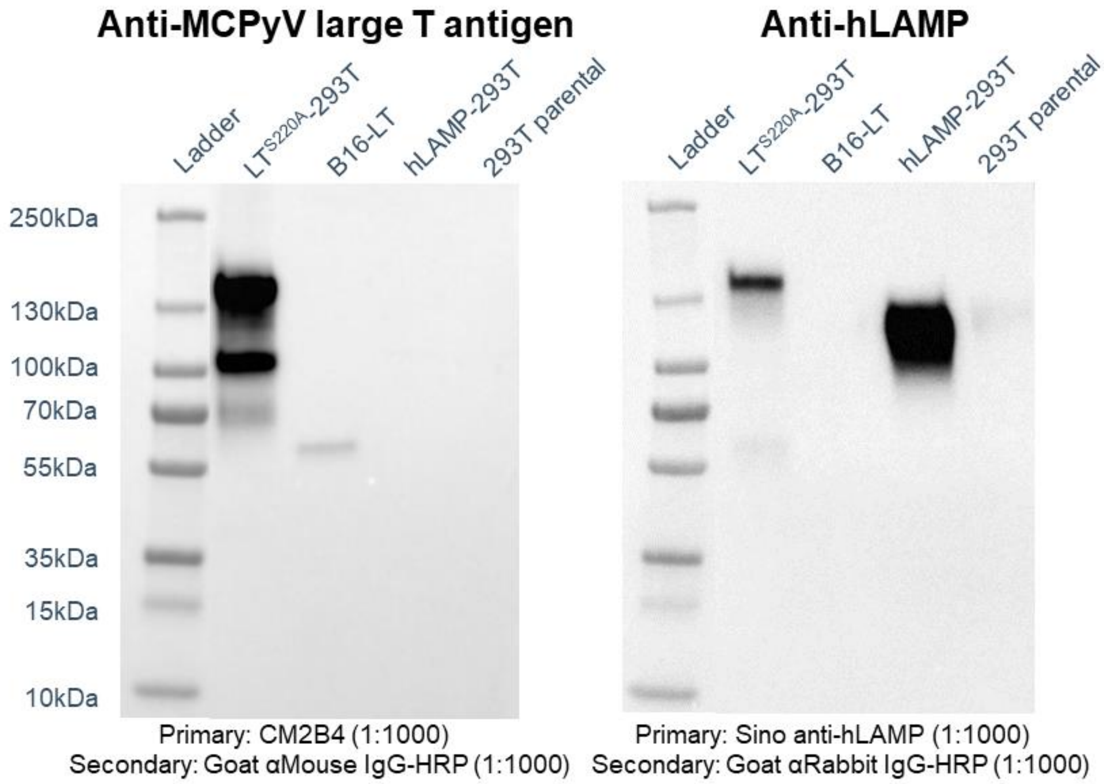

Supplementary Figure 1. Western blot validation of ITI-3000. 293T cells were transfected with 5µg of ITI-3000 using Lipofectamine 2000 (Thermo Fisher Scientific, Waltham, MA). Cells were transfected with 5µg of hLAMP pDNA or no pDNA as positive and negative controls, respectively. B16-LT cells were used as a positive control in the Western blot probed with anti-LT antibody. Cell extracts were prepared using RIPA buffer containing HALT protease inhibitor (Thermo Fisher Scientific) and electrophoresed using 4-15% TGX gels, then blotted onto 0.2 µM polyvinylidene difluoride (PVDF) membranes (Bio-Rad, Hercules, CA) using a Bio-Rad Turbo Blotter. The membranes were processed using the iBind Flex (Invitrogen, Waltham, MA) Western blot system, probing with either anti-McPyV-LT (clone CM2B4) (Santa Cruz Biotechnology, Dallas, TX) or anti-hLAMP1 (11215-RP02) (Sino Biological, Beijing, China) primary antibodies and goat anti-mouse IgG-HRP or goat anti-rabbit IgG-HRP (Southern Biotech, Birmingham, AL) secondary antibodies, respectively. The membranes were detected using Clarity Western ECL reagent (Bio-Rad), and images were acquired using the Syngene G-Box mini system and GeneSys software.

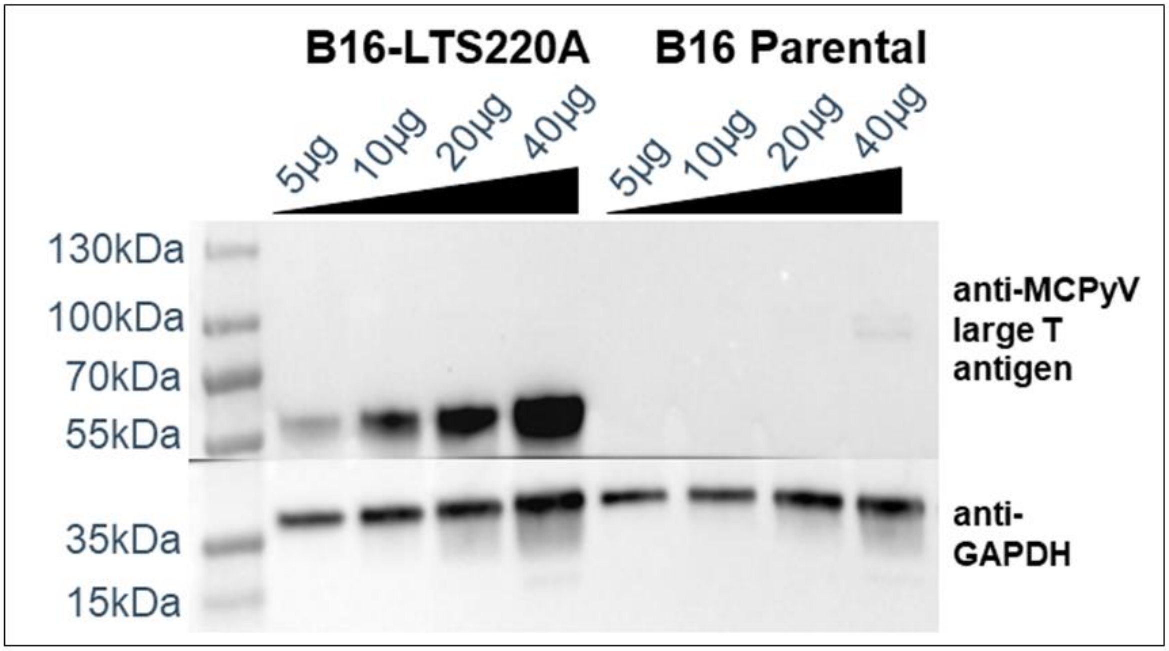

Supplementary Figure 2. Western blot validation of the B16-LT tumor cell line. Expression of LT^{S220A} in B16-LT cells was confirmed by Western blot. Cell extracts were prepared using RIPA buffer containing HALT protease inhibitor (Thermo Fisher Scientific) and electrophoresed using 4-15% TGX gels, then blotted onto 0.2 µM polyvinylidene difluoride (PVDF) membranes (Bio-Rad, Hercules, CA) using a Bio-Rad Turbo Blotter. 5µg, 10µg, 20µg, or 40µg of cell extract (either B16-LT or parental B16F10 cells) was used for Western blot analysis. The membranes were processed using the iBind Flex (Invitrogen, Waltham, MA) Western blot system. The anti-MCPyV LT primary antibody (clone CM2B4, Millipore Sigma) was used at 1:1000, and goat anti-mouse IgG-HRP was used at 1:1000 as a secondary antibody. The anti-GAPDH antibody (clone GA1R, ThermoFisher) was used as a loading control at 1:500 and goat anti-mouse IgG-HRP was used at 1:1000 as a secondary antibody. The membranes were detected using Clarity Western ECL reagent (Bio-Rad), and images were acquired using the Syngene G-Box mini system and GeneSys software. The transduced B16-LT tumor line shows expression of the LT antigen at the expected size, while the parental B16F10 melanoma line does not**.**

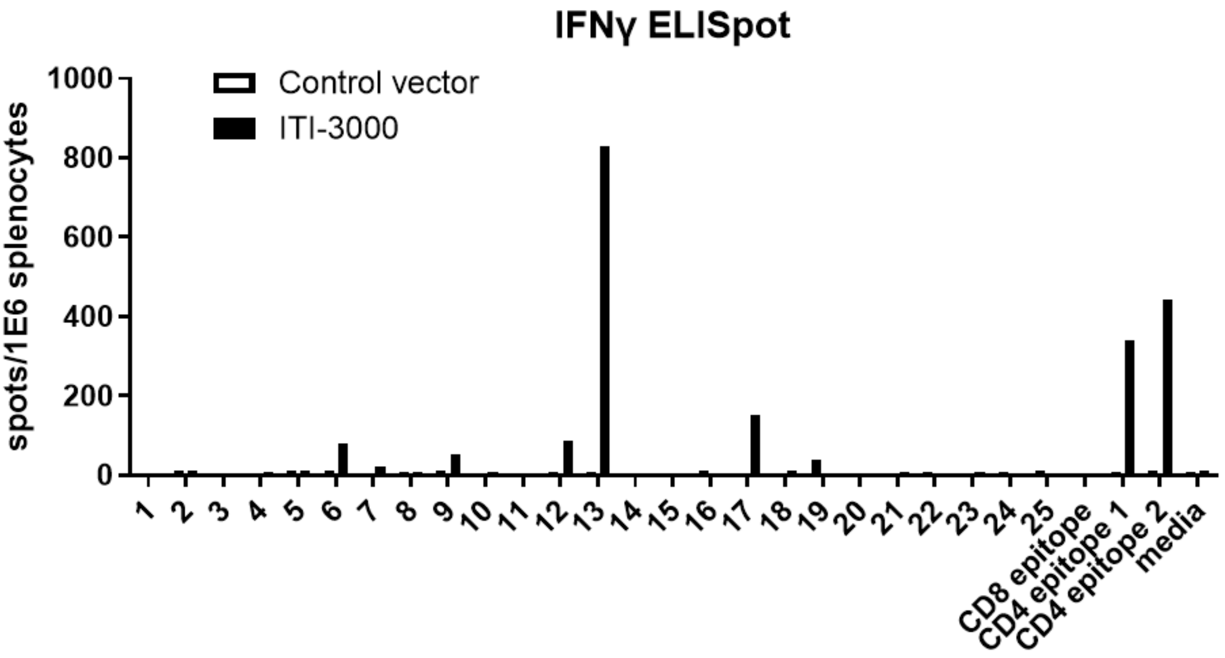

Supplementary Figure 3. Immune responses to individual peptides in peptide pool 2. Experimental design shown in Figure 2A. C57BL/6 mice were vaccinated four times, weekly, with 40µg of ITI-3000 or control vector via intradermal injection followed by electroporation. Mice were euthanized fourteen days following the final vaccination, and antigen-specific IFNγ peptide recall responses were evaluated in splenocytes by ELISpot. Individual peptides from peptide pool 2 (25 total peptides) were used to stimulate splenocytes. Media alone was used as a negative control. Previously identified CD4 and CD8 T cell epitopes for C57BL/6 mice were also tested. Data is represented as spot-forming units (SFU) per 1E6 splenocytes.

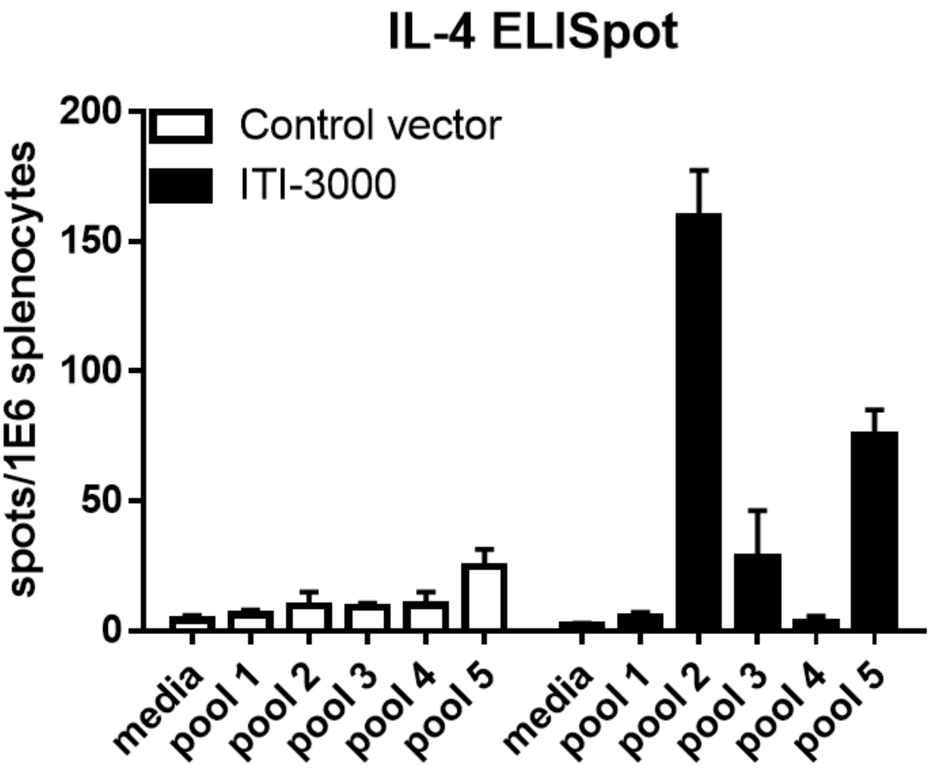

Supplementary Figure 4. IL-4 ELISpot data after vaccination with ITI-3000. Experimental design shown in Figure 2A. C57BL/6 mice were vaccinated four times, weekly, with 40µg of ITI-3000 or control vector via intradermal injection followed by electroporation. Mice were euthanized fourteen days following the final vaccination, and antigen-specific IL-4 peptide recall responses were evaluated in splenocytes by ELISpot. An overlapping peptide library spanning the large T antigen of MCPyV (five separate peptide pools) was used as stimulation at 2ug/ml. Media alone was used as a negative control. Data is represented as spot-forming units (SFU) per 1E6 splenocytes. Representative of two separate experiments.

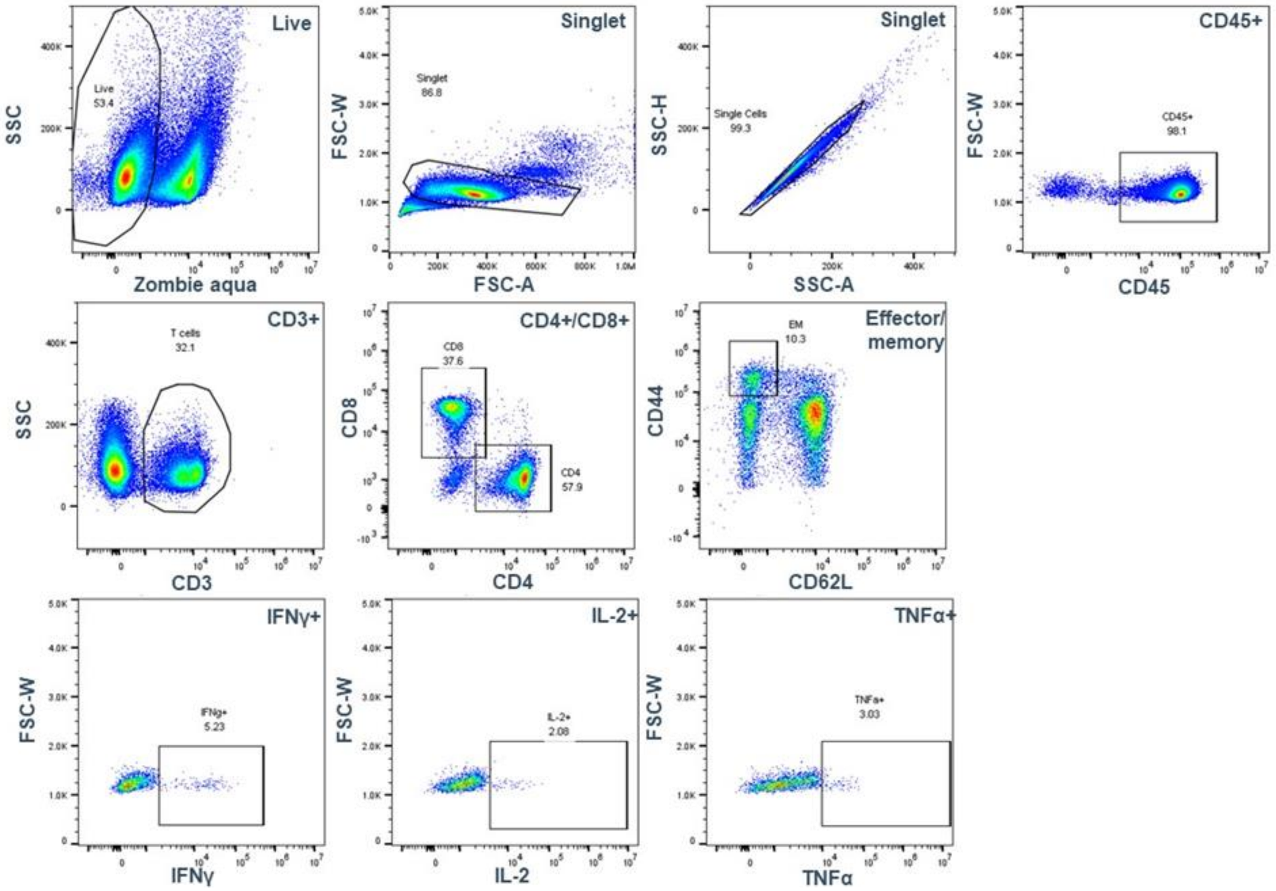

Supplementary Figure 5. Flow cytometry gating scheme for T cell panel. Gating scheme for flow cytometry panel “T cell panel” shown in Table S1.

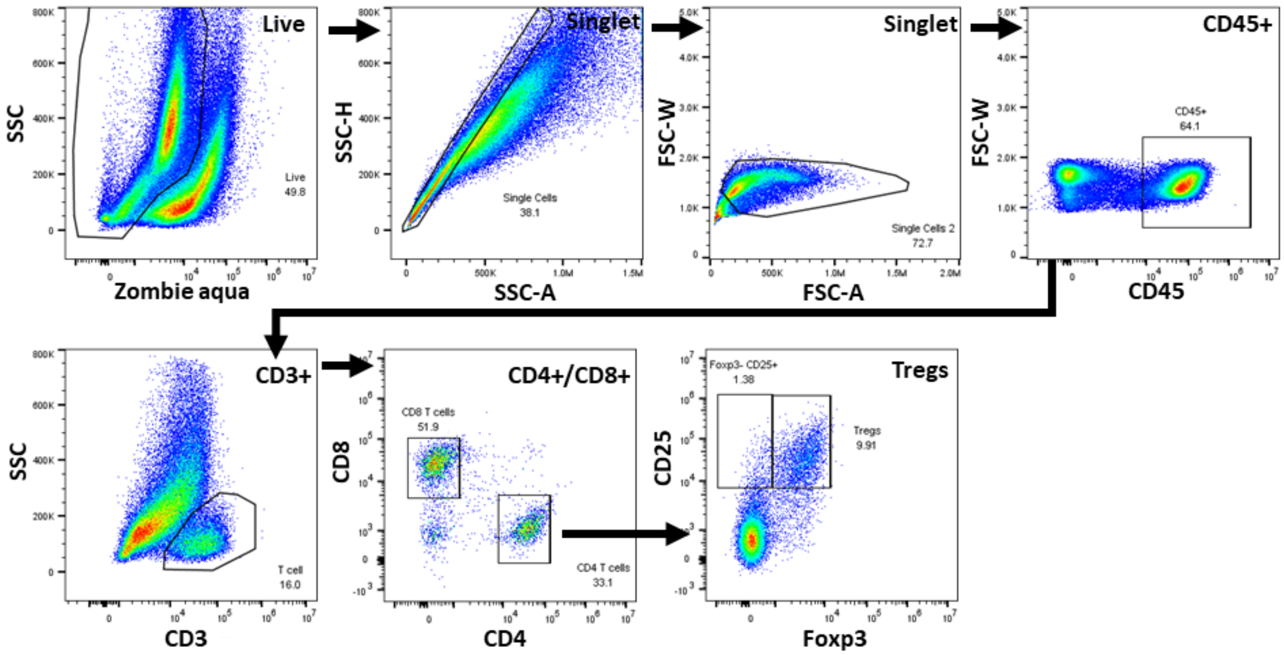

Supplementary Figure 6. Flow cytometry gating scheme for Treg panel. Gating scheme for flow cytometry panel “Treg panel” shown in Table S1.

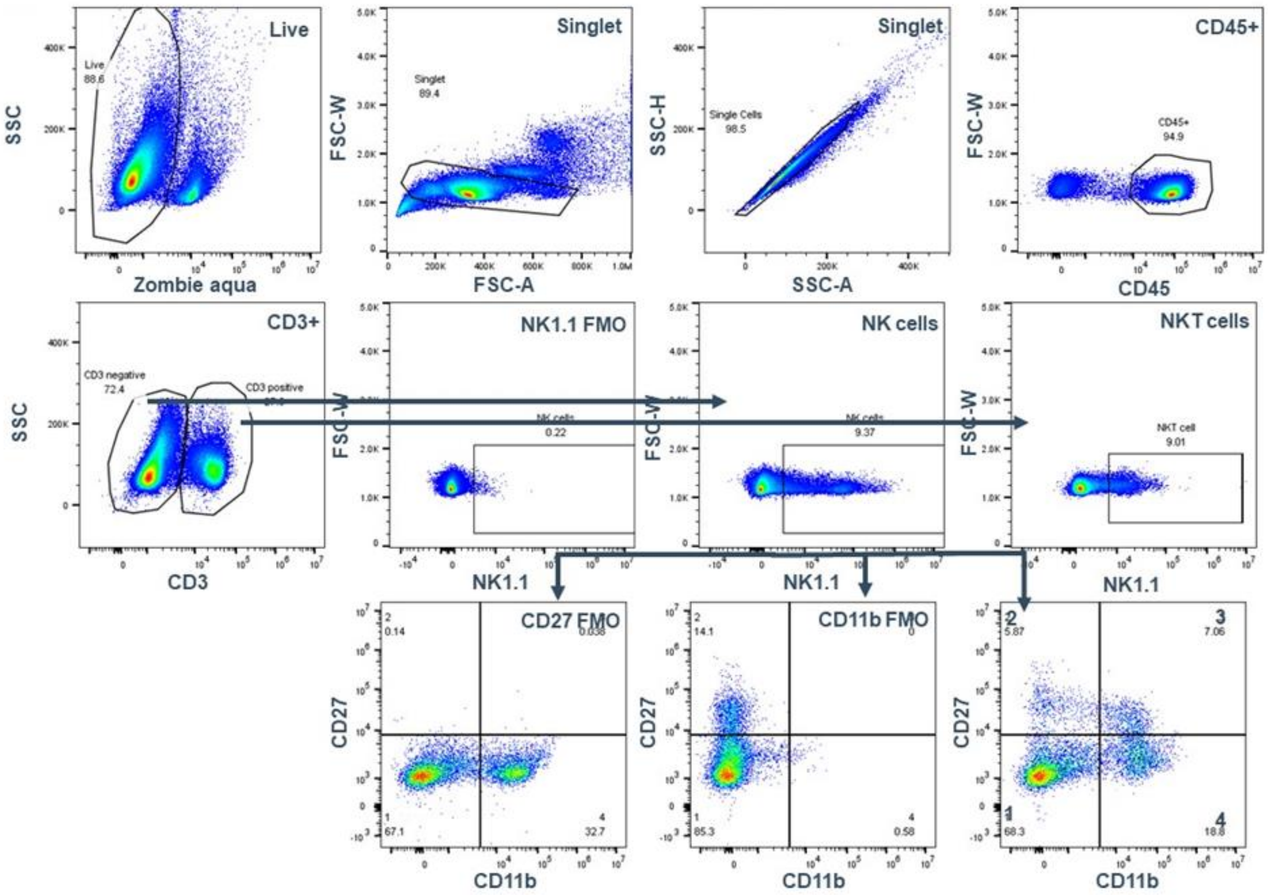

Supplementary Figure 7. Flow cytometry gating scheme for NK cell panel. Gating scheme for flow cytometry panel “NK cell panel” shown in Table S1.

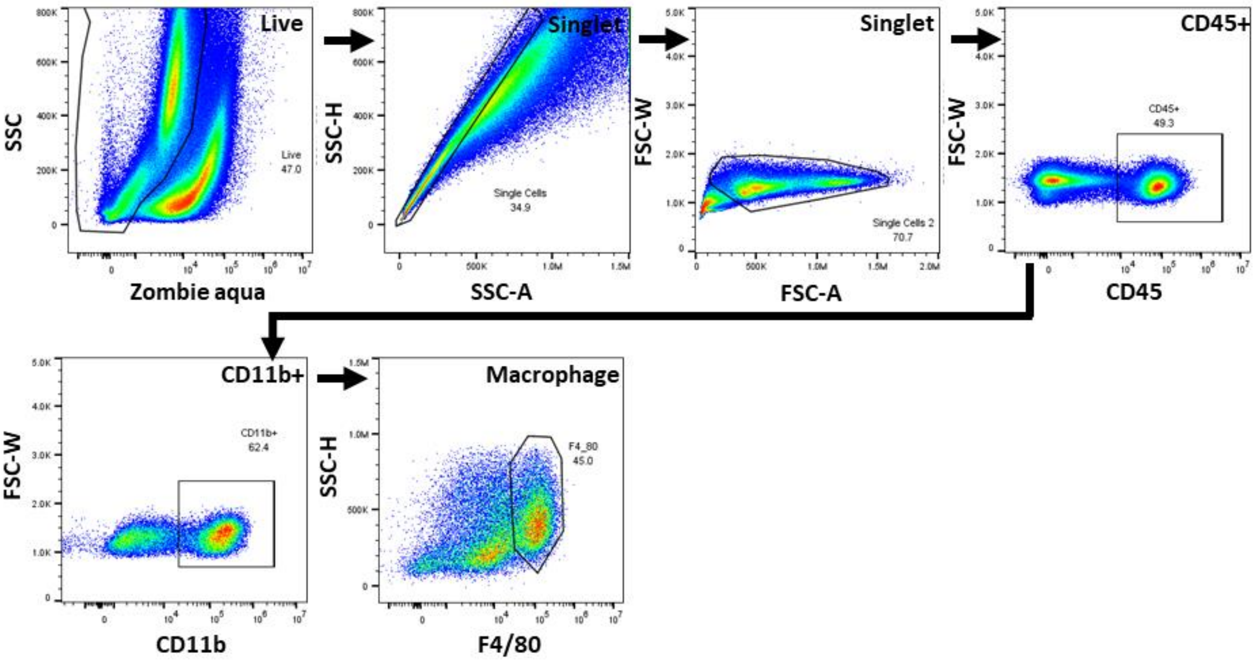

Supplementary Figure 8. Flow cytometry gating scheme for macrophage panel. Gating scheme for flow cytometry panel "Macrophage panel" shown in Table S1.

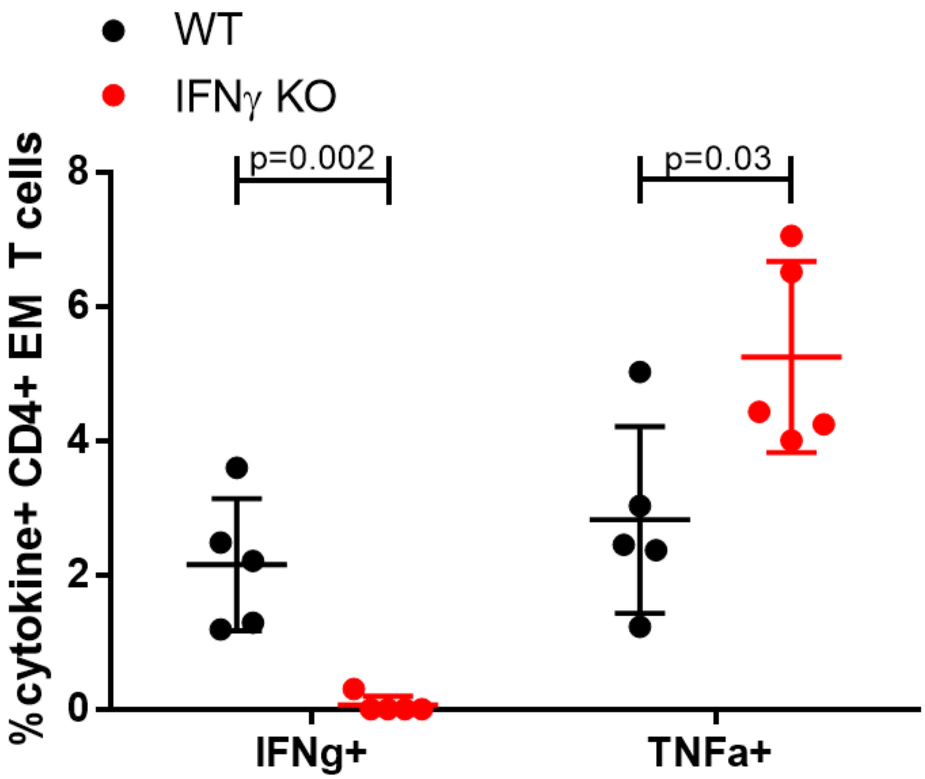

Supplementary Figure 9. IFNγ$^{-/-}$ mice show peptide recall response following vaccination with ITI-3000. C57BL/6 WT or IFNγ KO mice were vaccinated with ITI-3000 three times, weekly, by intradermal administration with electroporation in the ear pinnae. Thirteen days after the third vaccination, mice were euthanized and splenocytes were harvested. 9E5 live splenocytes were plated per well in the presence of brefeldin A, monensin, and MCPyV-LT peptide pool 2 (2µg/ml). Each splenocyte sample was also plated with no added stimulation as a no-peptide control. After five hours of incubation, cells were washed, stained with fluorochrome-conjugated antibodies from the "T cell panel," and analyzed using a CytoFLEX flow cytometer and FlowJo and GraphPad Prism 6 software. Data was graphed as percent cytokine-positive effector/memory CD4 T cells (live, singlet, CD3^{+}, CD4^{+}, CD44^{+}, CD62L^{lo}).

T cell panel				Treg panel				NK cell panel				Macrophage panel			
Fluorochrome	Marker	Vendor	Clone	Fluorochrome	Marker	Vendor	Clone	Fluorochrome	Marker	Vendor	Clone	Fluorochrome	Marker	Vendor	Clone
FITC	CD4	Biolegend	GK1.5	FITC	CD3	Biolegend	17A2	FITC	CD3	Biolegend	17A2	FITC	CD11b	Biolegend	M1/70
PE	IL-2	Biolegend	JES6-5H4	PerCP/Cy5.5	CD8α	Biolegend	53-6.7	PE	CD27	Biolegend	LG.3A10	APC-FIRE 750	CD45	Biolegend	30-F11
PerCP/Cy5.5	CD8α	Biolegend	53-6.7	PE-Cy7	CD25	Biolegend	PC61	PE-Cy7	NK1.1	Biolegend	PK136	BV421	F4/80	Biolegend	BM8
PE-Cy7	CD44	Biolegend	IM7	APC	CD4	Biolegend	GK1.5	APC	CD11b	Biolegend	M1/70	Zombie aqua		Biolegend	
APC	TNFα	Biolegend	MP6-XT22	APC-FIRE 750	CD45	Biolegend	30-F11	APC-FIRE 750	CD45	Biolegend	30-F11				
Alexa Fluor 700	CD3	Biolegend	17A2	BV421	Foxp3	Biolegend	MF-14	BV421	IFNγ	Biolegend	XMG1.2				
APC-Fire 750	CD45	Biolegend	30-F11	Zombie aqua		Biolegend		Zombie aqua		Biolegend					
BV421	IFNγ	Biolegend	XMG1.2												
BV605	CD62L	Biolegend	MEL-14												
Zombie aqua		Biolegend													

Supplementary Table 1. Flow cytometry panels. Fluorochromes, markers, vendors, and antibody clones for the T cell panel, Treg panel, NK cell panel, and macrophage panel.
